# Report of the Diseases of Edinburgh for April, 1809

**Published:** 1809-06

**Authors:** John Roberton


### *Report of the Diseases of Edinburgh for April*, 1809.
### By John Roberton, M. D.

The weather, for the first few days of the month, tho' cold, was clear, and tolerably pleasant. About the end of the first third of it, however, still continuing cold, it became cloudy, and was immediately followed by a general and very heavy fall of snow. This soon dissolved, and, in a few days, we were at once assailed by a fall of rain, hail, and snow, accompanied by a tremendous storm of wind. This sort of weather, though in a rather milder degree, continued till the beginning of the last third of the month, when the wind, which till then had blown almost constantly from the East, shifted to the South-West, and we had a few very pleasant days. This, however, was again succeeded by a heavy fall of rain, with the wind again from the East, continuing almost incessantly for two or three days. From this till the end of the month, the weather continued moist, but upon the whole tolerably pleasant.

About the beginning of the month, though the barometer stood high, it fell considerably in a few days, almost immediately previous to the fall of snow. After this, it again rose, and independently of the violent weather which followed it, still continued almost stationary; nor did it fall in any considerable degree till within the last few days of the termination of the month.

The thermometer altered very frequently during the month, but the extent of its changes were not considerable. It, in general, stood from 35 to 46 of Fahrenheit's scale.

The diseases which, during this period, have prevailed in greatest severity have been fevers, croup, cynanche tonsillaris, catarrh, rheumatism, and pneumonia.

The fevers formerly mentioned, still continue very bad, and seem now of a more contagious nature than they have been for several months. I have so frequently remonstrated upon this subject, and on the propriety and even absolute necessity of employing some means rather for the prevention than merely for the cure of such complaints, that I believe the subject has now become exhausted; I cannot, however, suppress my indignation at seeing so many valuable lives annually sacrificed, when, even by a twentieth part of the attention which is daily devoted to the accomplishment of objects, comparatively speaking, of no moment, ment, they might be preserved to their families and to their country.

Several cases of croup, among children, have occurred in practice. The method now generally adopted, in this place, of giving large doses of calomel in this disease, seems to answer our expectations; at least, it certainly is the best practice with which we are at present acquainted, and we therefore adopt it, till our further investigations point out some plan of treatment still more effectual.

Cynanche tonsillaris and catarrh still continue to prevail very extensively: indeed, they do not seem to have at all abated in their severity since last Report. The ridiculous custom, as formerly mentioned, of people throwing off part of their winter dress whenever a good day occurs, certainly protracts these complaints much longer than they would otherwise remain.

Rheumatism, both acute and chronic, seems still very general; probably, in a great measure, occasioned by the same cause which produces cynanche and catarrh. Some of the cases of this disease, which I have lately seen, were combined with fever of considerable severity. In some of them, the fever seemed somewhat similar to the typhus mentioned above, and in others it partook of the inflammatory cast.

The disease, in an uncombined state, was in general easily removed by diaphoretic medicines, and in some cases, in addition to these, it was necessary for their removal to apply a blister over the affected part. When the inflammatory diathesis greatly prevailed, blood-letting at once removed it as well as the pain, even without the use of any other application, or the necessity for the administration of any internal medicine.

I am extremely sorry to find that the inflammatory complaints, particularly pneumonia, have recurred with considerable violence; even with greater than during the whole winter. The attack of pneumonia has been very sudden, and its termination much more rapid as well as more frequently fatal, than I ever recollect to have seen it. We have been unfortunate enough to witness the apparently most healthy and active individuals seized with this complaint, and carried off in the course of from twenty to thirty, or thirty-six hours.

Under these circumstances, we have been obliged, in our treatment, to be uncommonly active, and the great and frequent bleedings which we have found it necessary to employ, are almost incredible. Even with our greatest activity activity in this way, with, at the same time, the administration of saline cathartics and repeated blisters over the affected part, we have often found it impossible to arrest the violence of the complaint, and the patients have consequently died. Still, however, this treatment, comparatively speaking, has been very successful; and it is through it alone that we are now daily in the habit of saving the lives of many valuable individuals.

Athough complaints of the generative organs, perhaps in the strict sense of the word, ought not to be introduced in a monthly Report, I cannot avoid taking notice of the immense number of cases of seminal emissions, which have lately come under my observation. This disease, which I now find to be of the most common occurrence, not being understood, and of course no rational or at all successful method of treatment being adopted for its removal, has ever been conceived scarcely to rank among the list of diseases. The scenes of misery, however, which have lately presented themselves to me, evidently caused by this alone, put to defiance all the powers of language to give the most distant idea of their extent.

I have long since irrefutably proved that the very worst cases of gleet may be cured by the *well regulated use* of cantharides used internally.* I have since, by the same medicine, been equally successful in the removal of seminal emissions, and though, in most instances, the cure has been much more tedious than in the former, yet in the sequel it has been equally decisive. On this subject, however, I shall not enlarge at present, but reserve, for a few weeks, what I principally have to advance, when I shall introduce it in my work on the Anatomy, Physiology, Pathology, and Practical Treatment of the Generative Organs, now nearly ready for the press.

*No. 4, St. James's Street.*

---

In my Report for February last, I made a few observations respecting the propriety of adopting some rational measure for the purpose of ascertaining the nature, and thereby, if possible, preventing the ravages which hydrophobia is supposed occasionally to make in different parts of the world.

In drawing up these observations, I trust I was actuated by

---

* See my Practical Treatise on that subject.

by the most disinterested and purest of motives. I considered myself as attempting, in my official capacity, to discharge a part of that duty to the public which it has a right to expect in deeds rather than in words, from every member of my profession, and especially from those who profess to devote some portion of their time and opportunities to humane investigation and charitable purposes. It ought to be remembered, that this duty consists not only in the more painful and humiliating office of constantly witnessing, or faithfully watching over our familiar acquaintances or our dearest friends, too often prematurely hurried to "that country from whose bourne no traveller returns," but in *preventing* the accession of those dreadful diseases which are the causes of such calamities.

That the subject of the inquiry to which I then alluded, and which I now resume, is of the very greatest importance, none, I believe, will attempt to question; and extremely imperfect as the medical art in general is, there is certainly no department of it involved in such obscurity as the disease termed hydrophobia. This circumstance, in addition to its dreadful nature, urged me strongly to make my previous proposal. In short, so ignorant are people in general of its real nature, that I believe, had we made no pretentions to account for it at all, but merely been directed in our mode of attempting the relief of its symptoms by an unprejudiced and close attention to the exact nature of such appearances as we imagine to indicate its existence, we certainly might have been much nearer the truth upon the subject, and could not have been farther from it than we are at the present moment.

That which renders the necessity of such an inquiry the more pressing, is the knowledge which in conversation every one assumes respecting its real nature; so much indeed, that even were the profundity of their knowledge candidly questioned on the subject, they would conceive themselves indifferently treated. Yet it is strange, that we seldom find two persons who exactly agree in opinion respecting the real nature of the disease. And this only shows that the more complicated and unaccountable a subject is, and the greater variety of ways there are of accounting for its effects, the more imperfect are our ideas respecting it, and consequently the further are we from a knowledge of its real nature. But to return.

It would however appear, that my extremely imperfect but willing attempt, to call the attention of some of my respectable, benevolent, and more powerful fellow-citizens zens to those acts of charity and of humanity, which they are in general so ready to afford, (for my remarks on that subject were in an especial manner addressed to them) is not likely to succeed on the present important occasion. But let it be understood, that I principally ascribe this tardiness of exertion entirely to my want of power of making myself fully understood by them, and neither to their want of willingness to come forward in the cause of humanity and mercy, nor to the want of importance in the subject of hydrophobia. Its almost paralyzing name, at all times, strikes more terror in the public mind, than can arise from almost any other external evil. Death itself, in a more natural way, is almost always borne with composure and resignation; but the fear of being doomed to the accumulated sufferings which we have been, from the earliest period of our lives, taught to believe as inseparable from that disease, unmans and almost involuntarily makes the firmest of us, during its supposed prevalence, shrink with terror at the appearance of the most puny of the canine race.

I may observe, as an apology for my again introducing this subject, that of all the many faults I am beset with, it surely is not one of my most conspicuous ones to relinquish any point I may have adopted, in science or in life, until it has been proved to me either useless or hurtful. Nor, in the present instance, shall I feel either discomposed or at all mortified, though the attempts I may make to encourage a devellopement of this subject should be, in a great measure, left to myself. These, as I formerly mentioned, are so often, indeed so invariably, the reward given for those investigations, which alone are to be beneficial to the public, that he who starts as candidate in such undertakings, without making allowance for innumerable failures, in having his researches applied to immediate use, will, unless he at once proposes and executes the design, be disappointed and chagrined in every action of his life; will be shocked and soured at what will seem to him the idle pursuits of the unmeaning, unfeeling, and self-interested mass of mankind. Otherwise fortified, however, every act which bears with it the prospect, even at some distant period, when the spirit of party may be hushed, of preventing distress, or of softening the pillow in the hour of affliction, will yield him delight which none but those devoted to such tasks can feel, and to which none but those devoted to such, or similar tasks, deserve.

To return therefore, to the subject, I shall, till the 1st of July next, at No. 12, Prince's Street, to which I shall remove before that period, receive Essays or Observations, (post paid,) on the subject of Hydrophobia, marked with the author's seal, accompanied by a separate paper, sealed also with the same seal, and enclosing the name of the author. I shall then select a proper committee to examine these Essays, some time within a month after the above period, and, according to their decision, shall award a small honorary medal, with an appropriate inscription; no paper enclosing the name of the unsuccessful candidates being examined, but shall be returned when desired.

*No. 4, St. James's Street.*

